# Darcy Forchhiemer imposed exponential heat source-sink and activation energy with the effects of bioconvection over radially stretching disc

**DOI:** 10.1038/s41598-024-58051-5

**Published:** 2024-04-04

**Authors:** K. M. Nihaal, U. S. Mahabaleshwar, S. W. Joo

**Affiliations:** 1 Department of Studies in Mathematics, Davangere University, Shivagangothri, Davangere, 577 007 India; 2 School of Mechanical Engineering, Yeungnam University, Gyeongsan, 38541 Korea

**Keywords:** Radially stretching sheet, Exponential heat source/sink, Darcy–Fochheimer, Activation energy, Bioconvection, Mathematics and computing, Nanoscale materials

## Abstract

The Darcy–Forchheimer model is a commonly used and accurate method for simulating flow in porous media, proving beneficial for fluid separation, heat exchange, subsurface fluid transfer, filtration, and purification. The current study aims to describe heat and mass transfer in ternary nanofluid flow on a radially stretched sheet with activation energy. The velocity equation includes Darcy–Fochheimer porous media effects. The novelty of this study is enhanced by incorporating gyrotactic microorganisms which are versatile and in nanofluid can greatly improve the thermal conductivity and heat transfer properties of the base fluid, resulting in more efficient heat transfer systems. Furthermore, the governing PDEs are reduced to ODEs via appropriate similarity transformations. The influence of numerous parameters is expanded and physically depicted through the graphical illustration. As the Forchheimer number escalates, so do the medium's porosity and drag coefficient, resulting in more resistive forces and, as a result, lowering fluid velocity. It has been discovered that increasing the exponential heat source/sink causes convective flows that are deficient to transport heat away efficiently, resulting in a slower heat transfer rate. The concentration profile accumulates when the activation energy is large, resulting in a drop in the mass transfer rate. It is observed that the density of motile microorganisms increases with a rise in the Peclet number. Further, the results of the major engineering coefficients Skin-friction, Nusselt number, Sherwood number, and Microorganism density number are numerically examined and tabulated. Also, the numerical outcomes were found to be identical to the previous study.

## Introduction

Now-a-days, the problems associated with boundary layer flow due to a stretching surface have gained prominence due to their practical utility in various industrial and technical workflows such as modern extrusion processes, extraction of copper wires, incremental sheet forming, and geothermal energy applications. The stretching rate and cooling rate have a major influence on the mechanical characteristics of the fluid that these systems demand as their output. As a result, the heat transfer properties of the stretching sheet have received considerable attention from researchers. Crane^1^ was the first to work on a stretching sheet problem where he explored the boundary layer flow over the surface. Later on, a significant number of researchers reviewed the stretching sheet problems in the context of various fluid models. Cortell^2^ addresses the numerical analysis for viscous flow and heat transfer across a non-linear stretching sheet. Reza-E-Rabbi^3^ explored MHD nanofluid flow over stretching sheet, the author illustrated comparative between Casson and Maxwell fluid over different flow fields. Jeelani and Abbas^4^ investigated suction and radiation effects over Maxwell hybrid nanofluid flow past a permeable inclined stretched sheet. MHD, radiation, and chemical reaction impacts over unsteady Casson nanofluid flow across a stretching sheet were investigated by Reza-E-Rabbi^5^. This article also explored the consequences of thermophoresis and Brownian motion over fluid flow. Considering the influence of radiation and magnetic field, Srinivasacharya and Kumar^6^ studied Casson flow on a stretching surface.

The solution to the flow problem was computed using artificial neural networks, and it was discovered that this neural network method was accurate, and the effectiveness of the solution improved as the number of neurons in the neural network expanded. Yousuf Ali^7^ examines the combined effects of Hall current and radiation on MHD nanofluid flow through a nonlinear stretching sheet. This study has found that when the effects of radiation and Hall current are combined, heat transfer is relatively higher than when only one of those effects is taken into account. With convective boundary conditions, Rafiqul Islam^8^ examined the heat and mass transport phenomena of a Casson nanofluid flow under the influence of MHD, heat source/sink, and chemical reaction.

The existence of a heat source or sink can have a substantial impact on the fluid flow and heat transfer characteristics. Several studies have been conducted to study the impacts of heat source/sink on various fluid flow scenarios, including MHD flow, nanoparticle aggregation, divergent/convergent channels, and non-Newtonian fluid flow^9–11^. These studies show that the existence of heat sources or sinks could manipulate temperature distribution, velocity profiles, and other flow parameters. Abbas et al.^12^ analyzed the implications of radiation and inclined MHD on nanofluid flow, taking into account exponential heat source/sink. Thermal radiation and chemical reactions are also investigated to determine the thermal and mass transfer properties of nanofluids. Khan et al.^13^ explored the Darcy–Forchhiemer flow of an Eyring–Powell nanofluid exposed to an exponential heat source/sink and gyrotactic microorganisms. The Cattaneo–Christov theory equation is utilized to highlight the heat and mass transfer phenomenon.

Flow owing to the Darcy–Forchheimer medium is a significant aspect of industrial applications such as geothermal energy production, catalytic converters, oil recovery processes, and gas turbines. There is a vast literature available on this topic such as: in the context of Darcy–Forchheimer model, Colak et al.^14^ investigated the Powell Eyring nanofluid over a stretching surface with bioconvection and artificial neural network. Mandal and Pal^15^ found dual solutions for the convective-MHD hybrid nanofluid in the Darcy–Forchheimer porous medium on a decreasing surface. Furthermore, the flow and heat transfer phenomenon were thoroughly investigated using stability analysis and entropy generation. Using the MHD Darcy–Fochheimer model, Joshi et al.^16^ investigated the effects of suction/injection and dissipation on hybrid nanofluid flow across a permeable stretched sheet. Moreover, for the blowing region, velocity profiles exhibit dual behavior as Forchhiemer and porosity parameters are increased. Paatanaik^17^ examined the combined impacts of Brownian and thermophoresis processes on nanofluid flow behavior across stretched surfaces, which also takes MHD, heat sources, and chemical reactions into account.

Activation energy is also used to investigate the nature of reactants and the effect of catalysts on reactions. Because activation energy is critical in boosting the rate of chemical processes, it is helpful in a variety of real-world applications such as match igniting, fire suppression, enzyme action, and many others. Certain reactions have been known to proceed slowly or not at all, even in the absence of a catalyst. Thus, an insignificant quantity of energy, known as activation energy, is required to start a chemical reaction. Babu and Sathian^18^ assessed the effects of activation energy and lower viscosity on water flow through CNTs. It was discovered that nonlinear fluctuation in viscosity increased the rate of fluid flow through carbon nanotubes. The bio-convective hybrid nanofluid flow across a riga plate in the presence of heat absorption and the activation energy was studied by A. Algehyne et al.^19^. However, many studies on activation energy have been carried out recently^20–22^.

Bioconvection is a phenomenon caused by the upward movement of microorganisms, which are denser than water, causing the topmost part of the liquid to become unstable and triggering the emergence of convection patterns. The movement of microorganisms in bioconvection can be purposefully regulated to produce certain outcomes, making it an effective way in biotechnological systems. Bioconvection has been established in studies to be essential for the development of medicine delivery systems, biofuel production, and biological polymer synthesis. Platt's reports^23^ were the first to discuss the term bioconvection, where he discovered Bioconvection patterns in Cells of Free-Swimming Organisms. Lui et al.^24^ investigated the numerical bio-convective evaluation for a rate-type nanofluid that was affected by unique slip characteristics and Nield thermal limitations. These recent studies^25–27^ have explored Bioconvection for different fluids with porous, non-linear radiation effects, and MHD. The below table shows the novel work in comparison with surveyed literatures (Table 1).

**Table 1:** Comparison of the current study with previously published papers.

Work	Darcy–Fochheimer model	Exponential heat source/sink	Activation energy	Bio-convection	Radial stretching sheet	Fluid type		
						Nano	Hybrid	Modified
Srinivasacharya and Kumar^28^	✗	✗	✗	✗	✓	✗	✗	✗
Raja et al.^29^	✓	✗	✓	✗	✓	✓	✗	✗
Waqas et al.^30^	✗	✓	✓	✓	✗	✓	✗	✗
Present study	✓	✓	✓	✓	✓	✗	✗	✓

**Figure 1:**
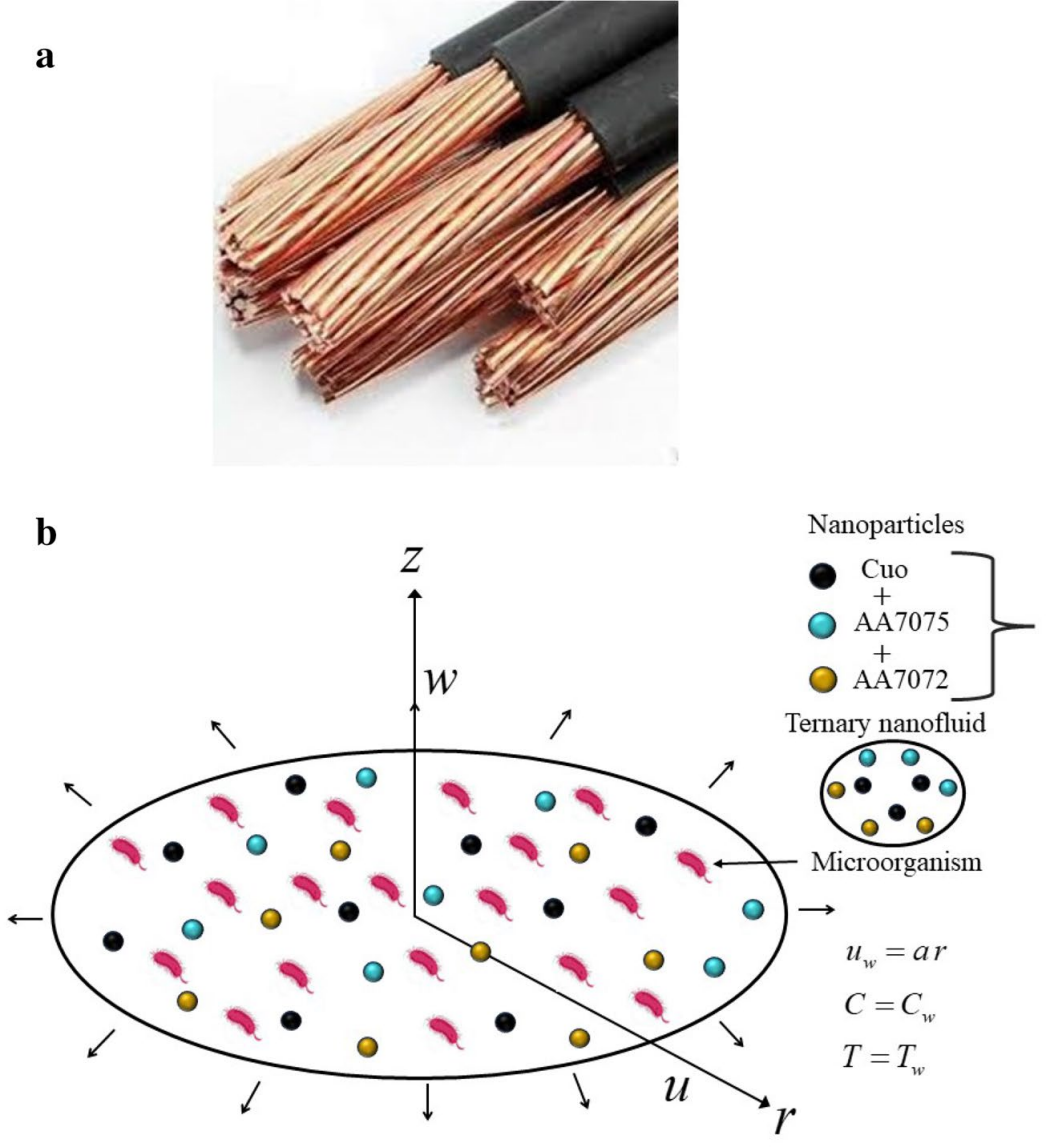
(**a**) Extraction of copper wire. Source (https://5.imimg.com/data5/SELLER/Default/2021/6/PA/DJ/GI/675420/copper-cable.jpg). (**b**) Schematic diagram.

High thermal conductivity, improved stability in base fluids, crystallinity, zero potential, and a huge surface area are the only benefits of ternary nanofluids. The most recent flow model has not yet been explored, as found by initial research. This study examines the Darcy–Forchheimer model with gyrotactic microorganisms of ternary nanofluid over a radially stretching sheet with activation energy. Radial stretching techniques have the potential to create unique copper wire products with specific geometries or performance requirements, such as those required for specialist electrical components or sophisticated manufacturing processes. This research helps in studying how usage of radially stretching sheets in the which makes copper wires suitable for electronic industries (Fig. 1a). The present flow problem is tackled with the bvp-4c algorithm. Plots are used to analyze the influence of distinctive parameters over velocity, temperature, concentration, and motile density profiles.

## Mathematical modeling

Consider Darcy–Forchheimer bio-convection flow of an incompressible nanofluid flow comprising nanoparticles $\left( {{\text{Cuo}},\,{\text{AA7072}},\,{\text{AA7075}}} \right)$ with two base fluids ethylene glycol and water across a disk at $z = 0$. The surface is elastic in nature and is stretching radially with velocity $u_{w} = ar$, $\left( {a > 0} \right)$ is constant as shown in Fig. 1b. Furthermore, exponential heat source/sink and activation energy are incorporated. $T_{w}$ and $T_{\infty }$, respectively represents the fluid's constant temperature at the wall and ambient temperature. The constant concentration and motile microorganisms on the sheet's wall surface are denoted by $C_{w}$ and $N_{w}$, respectively, and $C_{\infty }$, $N_{\infty }$ are the concentration and motile organisms at the far field.

The basic equations for the described problem can be expressed in the vector form as follows:

1 $$ \nabla \, \cdot \,{\vec{\text{V}}}\,{ = }\,{0,} $$

2 $$ \left( {\frac{{\partial {\vec{\text{V}}}}}{\partial t} + {\vec{\text{V}}} \cdot \,\nabla {\vec{\text{V}}}\,} \right) = \nu_{tnf} \nabla^{2} {\vec{\text{V}}} - \frac{{C_{b} }}{\sqrt K }{\vec{\text{V}}}\left| {{\vec{\text{V}}}} \right|, $$

3 $$ \left( {\rho Cp} \right)_{tnf} \left( {\frac{{\partial {\vec{\text{V}}}}}{\partial t} + {\vec{\text{V}}}\, \cdot \,\,\nabla {\text{T}}\,} \right) = K\nabla^{2} T + \left( {T - T_{\infty } } \right)Q^{*} {\text{exp}}\left( { - m\,\nu_{f}^{ - 0.5} a^{0.5} \,z} \right), $$

4 $$ \left( {\frac{{\partial {\text{C}}}}{\partial t} + {\vec{\text{V}}} \cdot \,\,\nabla {\text{C}}\,} \right) = D\nabla^{2} {\vec{\text{V}}} - k_{r}^{2} \left( {\frac{T}{{T_{\infty } }}} \right)^{n} e^{{\frac{{ - E_{a} }}{{K_{b} T}}\,\,}} \left( {C - C_{\infty } } \right), $$

5 $$ \left( {\frac{{\partial {\text{N}}}}{\partial t} + {\vec{\text{V}}} \cdot \,\,\nabla {\text{N}}\,} \right) = N{\tilde{\text{V}}} - D_{{\text{N}}} \nabla {\text{N,}} $$

Here, ${\vec{\text{V}}}$ is a function of $\left( {u,w} \right)$, and ${\tilde{\text{V}}} = \frac{{ - bW_{c} }}{{\left( {C_{w} - C_{\infty } } \right)}}\nabla {\text{C}}$. Under these essential stated assumptions, the PDEs form of the steady initial governing equations is expressed as per^28,31,32^.

6 $$ \frac{1}{r}\frac{\partial u}{{\partial r}} + \frac{u}{r} + \frac{\partial w}{{\partial z}} = 0, $$

7 $$ u\frac{\partial u}{{\partial r}} + w\frac{\partial u}{{\partial z}} = \nu_{tnf} \frac{{\partial^{2} u}}{{\partial z^{2} }} - \frac{{C_{b} }}{\sqrt K }u^{2} , $$

8 $$ u\frac{\partial T}{{\partial r}} + w\frac{\partial T}{{\partial z}} = \alpha_{tnf} \frac{{\partial^{2} T}}{{\partial z^{2} }} + \left( {T - T_{\infty } } \right)\frac{{Q^{*} }}{{\left( {\rho Cp} \right)_{tnf} }}{\text{exp}}\left( { - m\,\nu_{f}^{ - 0.5} a^{0.5} \,z} \right), $$

9 $$ u\frac{\partial C}{{\partial r}} + w\frac{\partial C}{{\partial z}} = D\frac{{\partial^{2} C}}{{\partial z^{2} }} - k_{r}^{2} \left( {\frac{T}{{T_{\infty } }}} \right)^{n} e^{{ - \frac{{E_{a} }}{{K_{b} T}}\,\,}} \left( {C - C_{\infty } } \right), $$

10 $$ u\frac{\partial N}{{\partial r}} + w\frac{\partial N}{{\partial z}} = \frac{{ - bW_{c} }}{{\left( {C_{w} - C_{\infty } } \right)}}\left[ {\frac{\partial }{\partial z}\left( {N\frac{\partial C}{{\partial z}}} \right)} \right] + D_{N} \frac{{\partial^{2} N}}{{\partial z^{2} }}, $$

with consistent boundary conditions

11 $$ \begin{aligned} & u = u_{w} ,\,w = 0,\,T = T_{w} ,\,C = C_{w} ,\,N = N_{w} \,at\,z = 0 \\ & u \to 0,\,T \to T_{\infty } ,\,C \to C_{\infty } ,\,\gamma \to \gamma_{\infty } \,as\,z \to \infty \\ \end{aligned} $$

where $\left( {u,w} \right)$ are the velocity components along $\left( {r,z} \right)$ axis. The term $\nu \left( {\mu /\rho } \right)$ and $\alpha \left( {k/\left( {\rho Cp} \right)} \right)$ denotes kinematic viscosity and thermal diffusivity, respectively. $C_{b}$ is drag coefficient, $K$ is the permeability of the porous medium, $Q^{*}$ is exponential heat source, $k_{r}$ denotes chemical reaction parameter, $E_{a}$ is activation energy component and $K$ is Boltzmann constant, and $\left( {T/T_{\infty } } \right)^{n} e^{{ - E_{a} /KT\,}}$ denotes modified Arrhenius function, $b$ represents chemotaxis constant, $D_{N}$ represents Microorganisms Diffusion coefficient, and $W_{c}$ represents maximum cell swimming speed. Thermo-physical properties of base fluids (water and ethylene glycol) and nanoparticles are assumed to be unchanged with temperature, and are listed in Table 2.

**Table 2:** Thermophysical properties of base fluids and nanoparticles at a temperature of 298.15 K and under normal circumstances see^33–35^.

Particles	$\rho \;({\text{kg}}\;{\text{m}}^{ - 3} )$	$Cp\;({\text{J}}\;{\text{kg}}^{ - 1} \;{\text{K}}^{ - 1} )$	$k\;({\text{W}}\;{\text{m}}^{ - 1} \;{\text{K}}^{ - 1} )$
${\text{H}}_{{2}} {\text{O}}$	997.1	4179	0.613
${\text{C}}_{{6}} {\text{H}}_{{2}} {\text{O}}_{{2}}$	1114	2415	0.252
${\text{CuO}}$	6500	533	17.65
${\text{AA7072}}$	2720	893	222
${\text{AA7075}}$	2810	960	173

The efficient properties of ternary nanofluid are stated below:

12 $$ \left. \begin{gathered} \mu_{tnf} = \frac{{\mu_{f} }}{{\left( {1 - \left( {\phi_{1} + \phi_{2} + \phi_{3} } \right)} \right)^{2.5} }},\,\rho_{tnf} = \left( {1 - \phi_{1} } \right)\left( {\left( {1 - \phi_{2} } \right)\left[ {\left( {1 - \phi_{3} } \right)\rho_{f} + \rho_{S3} \phi_{3} } \right] + \rho_{S2} \phi_{2} } \right) + \rho_{S1} \phi_{1} \hfill \\ \left( {\rho C_{p} } \right)_{tnf} = \left( {\frac{{\left( {\rho C_{p} } \right)_{S1} \phi_{1} }}{{\left( {\rho C_{p} } \right)_{f} }} + \left( {1 - \phi_{1} } \right)\left[ {\left( {1 - \phi_{2} } \right)\left( {\left( {1 - \phi_{3} } \right) + \frac{{\left( {\rho C_{p} } \right)_{S3} \phi_{3} }}{{\left( {\rho C_{p} } \right)_{f} }}} \right) + \frac{{\left( {\rho C_{p} } \right)_{S2} \phi_{2} }}{{\left( {\rho C_{p} } \right)_{f} }}} \right]} \right)\left( {\rho C_{p} } \right)_{f} , \hfill \\ k_{tnf} = \left( {\frac{{k_{S1} + 2k_{hnf} - 2\phi_{1} \left( {k_{hnf} - k_{S1} } \right)}}{{k_{S1} + 2k_{hnf} + \phi_{1} \left( {k_{hnf} - k_{S1} } \right)}}} \right)k_{hnf} ,\,\,k_{hnf} = \left( {\frac{{k_{S2} + 2k_{nf} - 2\phi_{2} \left( {k_{nf} - k_{S2} } \right)}}{{k_{S2} + 2k_{nf} + \phi_{2} \left( {k_{nf} - k_{S2} } \right)}}} \right)k_{nf} , \hfill \\ k_{nf} = k_{f} \left( {\frac{{k_{S3} + 2k_{f} - \left( {k_{f} - k_{S3} } \right)2\phi_{3} }}{{k_{S3} + 2k_{f} + \left( {\phi_{3} k_{f} - \phi_{3} k_{S3} } \right)}}} \right). \hfill \\ \end{gathered} \right\} $$

Here $\phi_{1}$, $\phi_{2}$, and $\phi_{3}$ represents volume fractions of individual nanoparticles. $\left( {tnf,\,hnf,\,nf} \right)$ are suffixes denoting ternary, hybrid, and nanofluid, respectively.

The suitable variables used for transformations are see^28^:

13 $$ \left. \begin{gathered} \eta = \frac{z}{r}{\text{Re}}^{1/2} ,\,\,u = u_{w} f^{\prime},\,\,w = u_{w} {\text{Re}}^{ - 1/2} \left( {\eta f^{\prime} - 2f} \right) \hfill \\ \theta \left( \eta \right) = \frac{{T - T_{\infty } }}{{T_{w} - T_{\infty } }},\,\,\chi \left( \eta \right) = \frac{{C - C_{\infty } }}{{C_{w} - C_{\infty } }},\,\,\gamma \left( \eta \right) = \frac{{N - N_{\infty } }}{{N_{w} - N_{\infty } }} \hfill \\ \end{gathered} \right\} $$

By utilizing Eq. (13), the system of Eqs. (6)–(10) and bcs. (11) are transformed into:

14 $$ \frac{{f^{\prime\prime\prime}}}{{A_{1} A_{2} }} + 2ff^{{\prime \prime }}  - Fr{\mkern 1mu} f^{{\prime 2}}  = 0, $$

15 $$ \left( {\frac{{A_{4} }}{{A_{3} }}} \right)\frac{1}{\Pr }\theta ^{\prime\prime} + 2f\theta ^{\prime} + Qs\,e^{( - m\eta )} \theta = 0, $$

16 $$ \chi ^{\prime\prime} + 2Scf\chi ^{\prime} - RcSc\left( {1 + \delta \theta } \right)^{n} e^{{\frac{E}{{ - \left( {1 + \delta \theta } \right)}}}} \chi = 0, $$

17 $$ \gamma ^{\prime\prime} - Pe\left[ {\chi ^{\prime\prime}\left( {\gamma + \omega } \right) + \gamma ^{\prime}\chi ^{\prime}} \right] + 2Lbf\gamma ^{\prime} = 0, $$

with

18 $$ \left. \begin{gathered} f^{\prime} = 1,\,\,f = 0,\,\,\theta = 1,\,\,\chi = 1,\,\,\gamma = 1, \hfill \\ f^{\prime} \to 0,\,\,\theta \to 0,\,\,\chi \to 0,\,\,\gamma \to 0. \hfill \\ \end{gathered} \right\} $$

The variables included in the equations are as follows:

$Fr = \frac{{C_{b} r}}{\sqrt K }$ is a Forchheimer number, $\Pr = \frac{\nu }{\alpha }$ is a Prandtl number, $Qs = \frac{Q*}{{a\left( {\rho Cp} \right)_{f} }}$ is an exponential heat source/sink parameter, $Rc = \frac{{K_{r}^{2} }}{r}$ is a reaction rate parameter, $Sc = \frac{{\nu_{f} }}{{D_{f} }}$ is a Schmidt number, $\delta = \frac{{T_{w} - T_{\infty } }}{{T_{w} }}$ is a temperature difference, $E$ is a activation energy, $Pe = \frac{{bW_{c} }}{{D_{N} }}$ is a Peclet number, $Lb = \frac{{\nu_{c} }}{{D_{N} }}$ is a Lewis number, ${\text{A}}_{{1}} = \left( {1 - \phi_{1} - \phi_{2} - \phi_{3} } \right)^{2.5}$, $$\begin{gathered} {\text{A}}_{2} = \left( {1 - \phi_{1} } \right)^{2.5} \left( {\left( {1 - \phi_{2} } \right)\left[ {\left( {1 - \phi_{3} } \right) + \frac{{\rho_{S3} }}{{\rho_{f} }}\phi_{3} } \right] + \frac{{\rho_{S2} }}{{\rho_{f} }}\phi_{2} } \right) + \frac{{\rho_{S1} }}{{\rho_{f} }}\phi_{1} ,\;A_{4} = \frac{{k_{tnf} }}{{k_{f} }}, \hfill \\ {\text{A}}_{3} = \left( {\frac{{\left( {\rho C_{p} } \right)_{S1} \phi_{1} }}{{\left( {\rho C_{p} } \right)_{f} }} + \left( {1 - \phi_{1} } \right)\left[ {\left( {1 - \phi_{2} } \right)\left\{ {\left( {1 - \phi_{3} } \right) + \frac{{\left( {\rho C_{p} } \right)_{S3} \phi_{3} }}{{\left( {\rho C_{p} } \right)_{f} }}} \right\} + \frac{{\left( {\rho C_{p} } \right)_{S2} \phi_{2} }}{{\left( {\rho C_{p} } \right)_{f} }}} \right]} \right). \hfill \\ \end{gathered}$$.

The physical quantities are given by,

19 $$ Cf = \frac{{\tau_{w} }}{{\rho_{f} u_{w}^{2} }},\;\tau_{w} = \left[ {\mu_{tnf} \frac{\partial u}{{\partial z}}} \right]_{z = 0} , $$

20 $$ Nu = - \frac{{rq_{w} }}{{k_{f} \left( {T_{w} - T_{\infty } } \right)}},\;q_{w} = - k_{tnf} \left( {\frac{\partial T}{{\partial z}}} \right)_{z = 0} , $$

21 $$ Sh = - \frac{{D_{f} rj_{w} }}{{\left( {C_{w} - C_{\infty } } \right)}},\;j_{w} = - \frac{1}{{D_{f} }}\left( {\frac{\partial C}{{\partial z}}} \right)_{z = 0} , $$

22 $$ Nh = - \frac{{rj_{n} }}{{D_{N} \left( {N_{w} - N_{\infty } } \right)}},\;j_{n} = - D_{N} \left( {\frac{\partial N}{{\partial z}}} \right)_{z = 0} , $$

From the above equations the Skin friction, Nusselt number, Sherwood number, and motile density are obtained as follows:

23 $$ {\text{Re}}^{1/2} Cf = \frac{f^{\prime\prime}\left( 0 \right)}{{A_{1} A_{2} }},\;{\text{Re}}^{ - 1/2} Nu = - \frac{{k_{tnf} }}{{k_{f} }}\theta ^{\prime}\left( 0 \right),\;{\text{Re}}^{ - 1/2} Sh = - \chi ^{\prime}\left( 0 \right)\;{\text{and}}\;{\text{Re}}^{ - 1/2} Nh = \gamma ^{\prime}\left( 0 \right) $$

## Methodology

This section discusses the methodology used to deduce solutions as well as code validation. The equations are initially modeled as PDEs and then transformed into ODEs using similarity variables. The "bvp-4c function" (a built-in package in MATLAB) is used to deduce the solution of Eqs. (14)–(17), as well as the BCs (18). With the aid of the "three-stage Lobatto IIIA formula", the "bvp4c function" employs a finite difference scheme and fourth-order accuracy. To achieve accurate results, the suitable boundary layer thickness, point depicting the far field $\eta_{\infty }$, and initial guess must be selected based on the parameters applied. To determine the model's solution, we convert the revised equations into a first-order system by introducing additional parameters. By utilizing these new parameters, the Eqs. (14)–(17) are reduced by following substitutions

$$ \left\{ {f = d_{1} ,f^{\prime} = d_{2} ,f^{\prime\prime} = d_{3} ,\theta = d_{4} ,\theta ^{\prime} = d_{5} ,\chi = d_{6} ,\chi ^{\prime} = d_{7} ,\gamma = d_{8} ,\gamma ^{\prime} = d_{9} } \right\} $$

The following MATLAB syntax is used for equations and BCs.

24 $$ d^{\prime}_{3} = - A_{1} A_{2} \left( {2d_{1} d_{3} - Fr\,d_{2}^{2} } \right), $$

25 $$ d^{\prime}_{5} = - \Pr \left( {2d_{1} d_{5} + Qs\,e^{( - m\eta )} d_{4} } \right), $$

26 $$ d^{\prime}_{7} = - Sc\left( {2d_{1} d_{7} - Rc\left( {1 + \delta d_{4} } \right)^{n} e^{{\frac{E}{{ - \left( {1 + \delta d_{4} } \right)}}}} d_{6} } \right), $$

27 $$ d^{\prime}_{9} = - \left( { - Pe\left[ { - Sc\left( {2d_{1} d_{7} - Rc\left( {1 + \delta d_{4} } \right)^{n} e^{{\frac{E}{{ - \left( {1 + \delta d_{4} } \right)}}}} d_{6} } \right)\left( {d_{8} + \omega } \right) + d_{9} d_{7} } \right] + 2Lbd_{1} d_{9} } \right), $$

and

28 $$ \left. \begin{gathered} d_{2} \left( 0 \right) = 1,\,d_{1} \left( 0 \right),\,d_{4} \left( 0 \right),\,d_{6} \left( 0 \right) = 1,\,d_{8} \left( 0 \right) = 1, \hfill \\ d_{2} \left( \infty \right) = 0,\,d_{4} \left( \infty \right) = 0,d_{6} \left( \infty \right) = 0,\,d_{8} \left( \infty \right) = 0\,.\, \hfill \\ \end{gathered} \right\} $$

**Figure 2:**
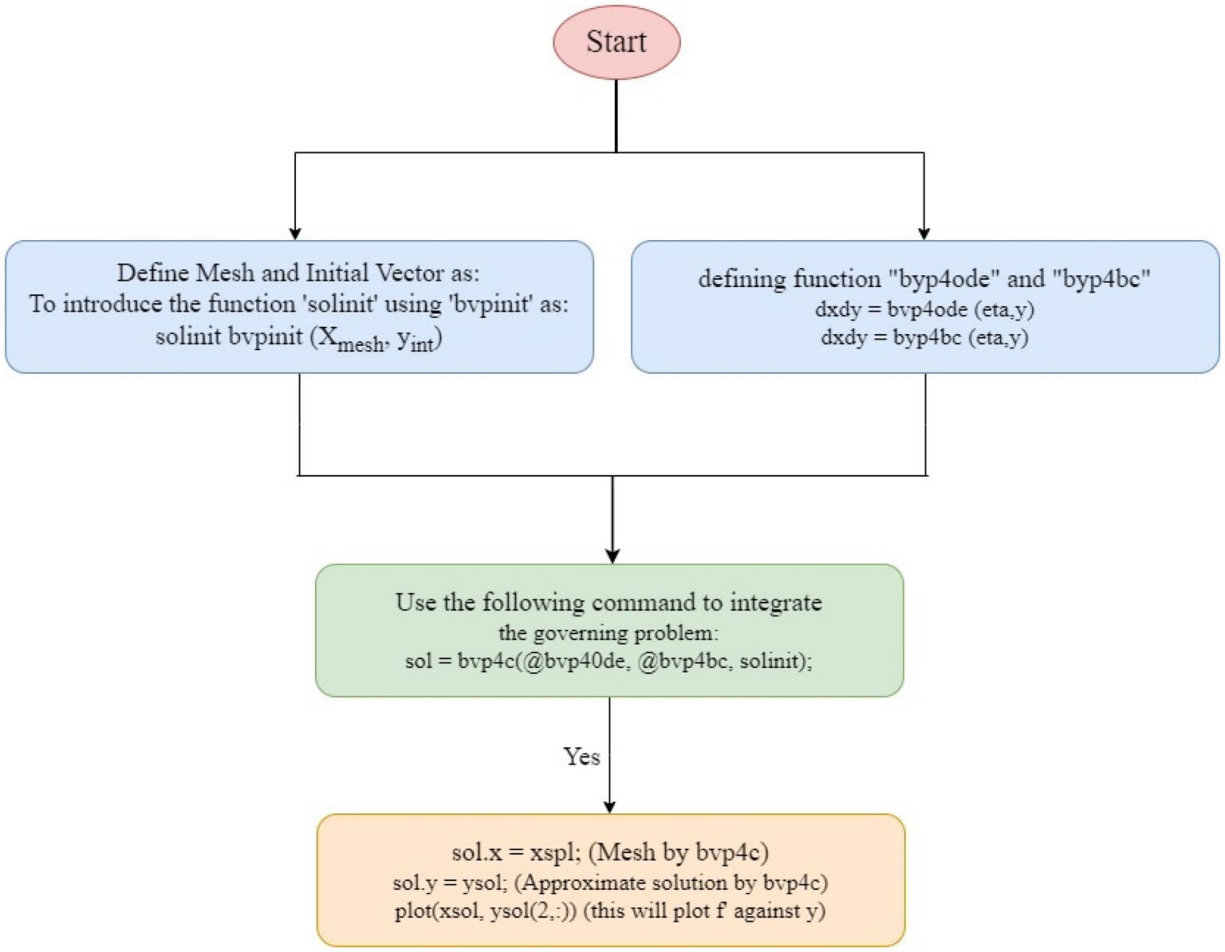
Flow chart of the problem.

The numerical solutions are then derived by feeding Eqs. (24) through (27) into the bvp-4c solver. The "sol" component is the outcome of bvp4c. "sol = bvp4c (@OdeBVP, @OdeBC, solinit)" provides the solver's syntax, which is made up of a couple of functions. The codes for Eqs. (24)–(27) are encoded in the ''@OdeBVP'' function. The BCs are defined using the ''@OdeBC" function (28). The ''solinit" function is used to code the initial mesh points and the initial solution approximation at those places. The solver will then execute which requires the substitution of the parameters $Fr = 0.2$, $\Pr = 6.3\left( {{\text{water}}} \right)/7.56\left( {\text{Engine oil}} \right)$, $Qs = 0.3$, $m = 0.01$, $\eta = 3$, $Sc = 0.6$, $Rc = 0.5$, $\delta = 0.1$, $E = 0.1$, $Pe = 0.5$, and $Lb = 0.5$ with characteristics listed in Table 1. and properties of ternary nanofluids. A graphical representation of the results achieved for each constraint by altering each parameter while keeping other parameters still and the results will be shown via graphs. Furthermore, the values of missing conditions are estimated to begin the process of finding the solution, and other parameters in Eqs. (24)–(27) are set to get the desired result. The solution is accepted after iterating and satisfying the conditions in Eq. (28) asymptotically. Figure 2 depicts the flow chart for determining the solution. We have taken the mesh size equal to 100. The convergence of the solution depends on the $\eta \to \infty$ values such as the values of the different parameters choosen by asymptotic convergence of the numerical outcome.

Furthermore, when the numerical outcomes were compared to those from previous studies (see Table 3), the results were quite matching.

**Table 3:** Values of $f^{\prime\prime}\left( 0 \right)$ when $M = 0$, $Fr = 1$ in the absence of $A_{1}$ and $A_{2}$.

Sl. No	Previous study $f^{\prime\prime}\left( 0 \right)$^36^	Present study $f^{\prime\prime}\left( 0 \right)$
1	1.17372	1.17328

## Results and discussion

This section of the work addresses the thorough analysis and physical interpretation of the momentum, temperature, concentration, and motile density profiles of the (water and ethylene glycol)-based $Cuo,\,\,{\text{AA7072}},\,\,{\text{AA7075}}$ ternary nanofluids. For various values of the relevant parameters encountered in the problem, the numerically generated results for the friction coefficient, heat and mass transfer coefficients, and motile density are plotted via graphs utilizing bvp4c solver. The current study's authors examined the model in two separate base fluid scenarios: water and engine oil.

Table 4 shows the results computed of Engineering coefficients limiting certain variables. The range of the effective used for the computational purposes are as follows: $0 \le Fr \le 0.6$, $0.1 \le Qs \le 0.7$, $0.1 \le Rc \le 0.7$, $0.5 \le E \le 2.0$, $0.5 \le Pe \le 2.0$, $0.3 \le Lb \le 0.9$. Table 3 shows the results computed of $f^{\prime\prime}\left( 0 \right)$, $\theta ^{\prime}\left( 0 \right)$, $\chi ^{\prime}\left( 0 \right)$, and $\gamma ^{\prime}\left( 0 \right)$ with limiting certain variables. From the table it is clear that upsurge in the value of $Fr$ increases surface drag coefficient $f^{\prime\prime}\left( 0 \right)$ and rate of heat transfer $\theta ^{\prime}\left( 0 \right)$ whereas drop in mass transfer $\chi ^{\prime}\left( 0 \right)$ and motile density $\gamma ^{\prime}\left( 0 \right)$ is observed. The rise in the $Qs$ has drop the thermal distribution by thickening of thermal boundary layer. Improvement in the values of $Sc$, $\delta$ and $Rc$ will increase the $\chi ^{\prime}\left( 0 \right)$ and $\gamma ^{\prime}\left( 0 \right)$ profiles. The decrease in $\chi ^{\prime}\left( 0 \right)$ is observed for rising values of $E$ but the $\gamma ^{\prime}\left( 0 \right)$ profile reduces due to decreased microorganism diffusivity in the fluid. The augmentation in the values of $Pe$ and $Lb$ increases $\gamma ^{\prime}\left( 0 \right)$ enhancing swimming rate of motile microorganisms. Upsurge in the values of $\Pr$ has elevated $f^{\prime\prime}\left( 0 \right)$ and $\theta ^{\prime}\left( 0 \right)$ profiles but opposite trend is witnessed in case of $\chi ^{\prime}\left( 0 \right)$ and $\gamma ^{\prime}\left( 0 \right)$ profiles.

**Table 4:** Computational values of Engineering coefficients $f^{\prime\prime}\left( 0 \right)$, $\theta ^{\prime}\left( 0 \right)$, $\chi ^{\prime}\left( 0 \right)$, and $\gamma ^{\prime}\left( 0 \right)$.

$Fr$	$Qs$	$m$	$Sc$	$Rc$	$\delta$	$E$	$Pe$	$Lb$	$\Pr$	$f^{\prime\prime}\left( 0 \right)$	$\theta ^{\prime}\left( 0 \right)$	$\chi ^{\prime}\left( 0 \right)$	$\gamma ^{\prime}\left( 0 \right)$
0										1.424490	1.017068	0.673278	0.593494
0.2										1.555241	1.001242	0.669746	0.583353
0.4										1.732649	1.984090	0.666212	0.573050
	0.1									1.555242	1.208986	0.669710	0.583332
	0.3									1.555241	1.001242	0.669746	0.583353
	0.5									1.555240	1.891208	0.669767	0.583365
		0.01								1.555241	1.001242	0.669746	0.583353
		0.02								1.555242	1.010792	0.669745	0.583353
		0.03								1.555242	1.020021	0.669743	0.583352
			0.2							1.555241	1.001242	0.364635	0.503481
			0.4							1.555241	1.001242	0.533298	0.542939
			0.6							1.555241	1.001242	0.669746	0.583353
				0.1						1.555241	1.001242	0.452514	0.508733
				0.3						1.555241	1.001242	0.573485	0.547933
				0.5						1.555241	1.001242	0.669746	0.583353
					0.1					1.555241	1.001242	0.669746	0.583353
					0.2					1.555241	1.001242	0.670673	0.583953
					0.3					1.555241	1.001242	0.671537	0.584509
						0.5				1.555241	1.001242	0.593133	0.555281
						1.0				1.555241	1.001242	0.597482	0.530824
						1.5				1.555241	1.001242	0.600471	0.514787
							0.5			1.555241	1.001242	0.669746	0.583353
							1.0			1.555241	1.001242	0.669746	0.634450
							1.5			1.555241	1.001242	0.669746	0.696265
								0.3		1.555241	1.001242	0.669746	0.508990
								0.5		1.555241	1.001242	0.669746	0.583353
								0.7		1.555241	1.001242	0.669746	0.732241
									6.3	1.555241	1.001242	0.669746	0.583353
									7.56	1.555242	1.215377	0.669667	0.583309

**Figure 3:**
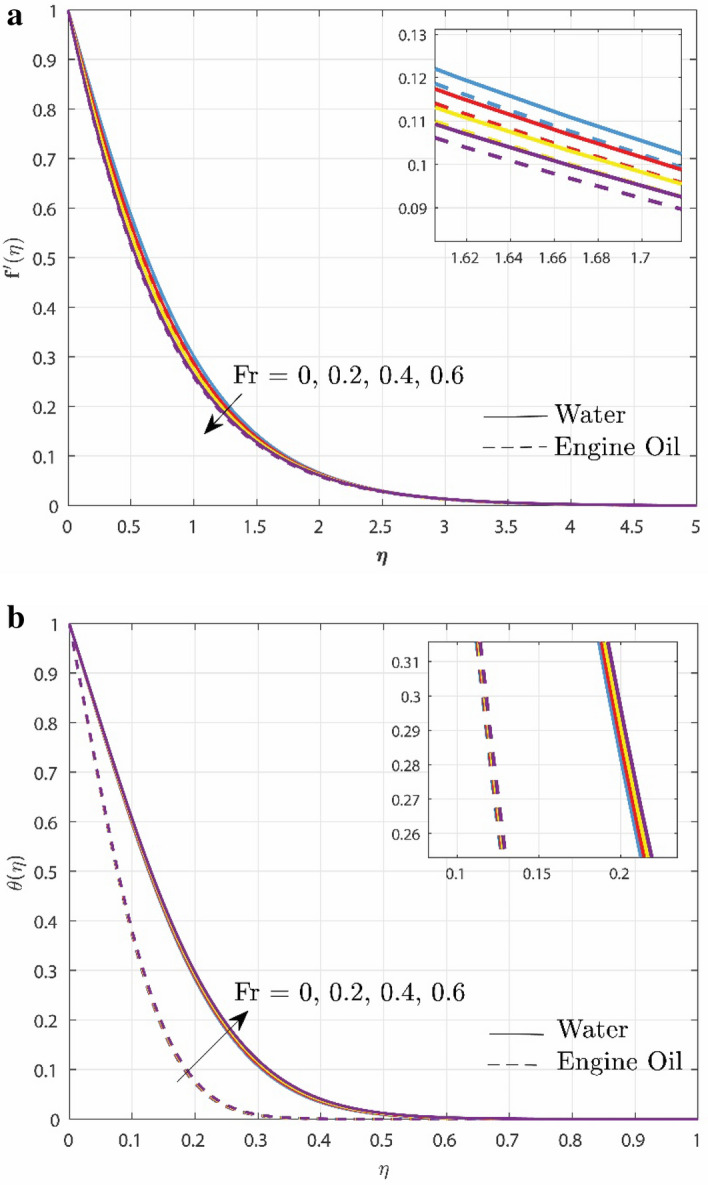
(**a**) Effect of $Fr$ over velocity profile. (**b**) Effect of $Fr$ over temperature profile.

Figure 3a,b visualizes the influence of Forchheimer number $\left( {Fr} \right)$ over velocity and temperature profile, respectively. A larger value of $Fr$ number displays a drop in fluid’s velocity whereas the opposite nature is seen in the temperature field for both cases i.e., water and engine oil. The explanation for this behavior is that the inertia of the porous media adds more resistance to the fluid flow, causing the fluid to move at a slower rate with a lower temperature. Due to their physical properties, in the velocity profile, the decline in Engine oil is more than in water but the temperature rise is more in water when compared to engine oil.

**Figure 4:**
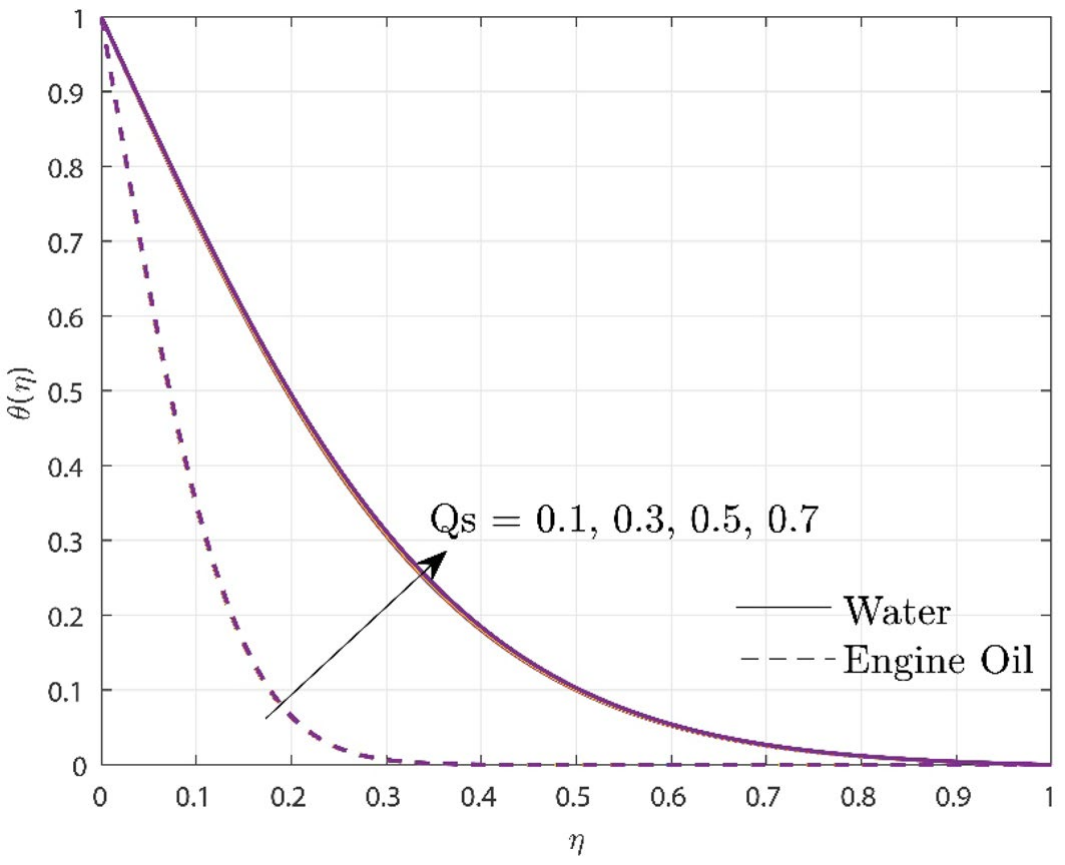
Upshot of $Qs$ over temperature profile.

Figure 4 describes the enhancement in the temperature profile $\theta \left( \eta \right)$ under the impact of exponential heat source/sink parameter $\left( {Qs} \right)$. It is obvious from the figure that increase in $Qs$ promotes temperature rises. The temperature rise is prompted by the transfer of heat to the system via the heat source's internal fluctuations. Due to varied thermal conductivity of two base fluids, the temperature elevation is higher in water rather than in engine oil. The implementation of exponential heat sources/sinks can optimize heat distribution and energy utilization in industrial processes like nuclear reactors, steel manufacturing, and many more.

**Figure 5:**
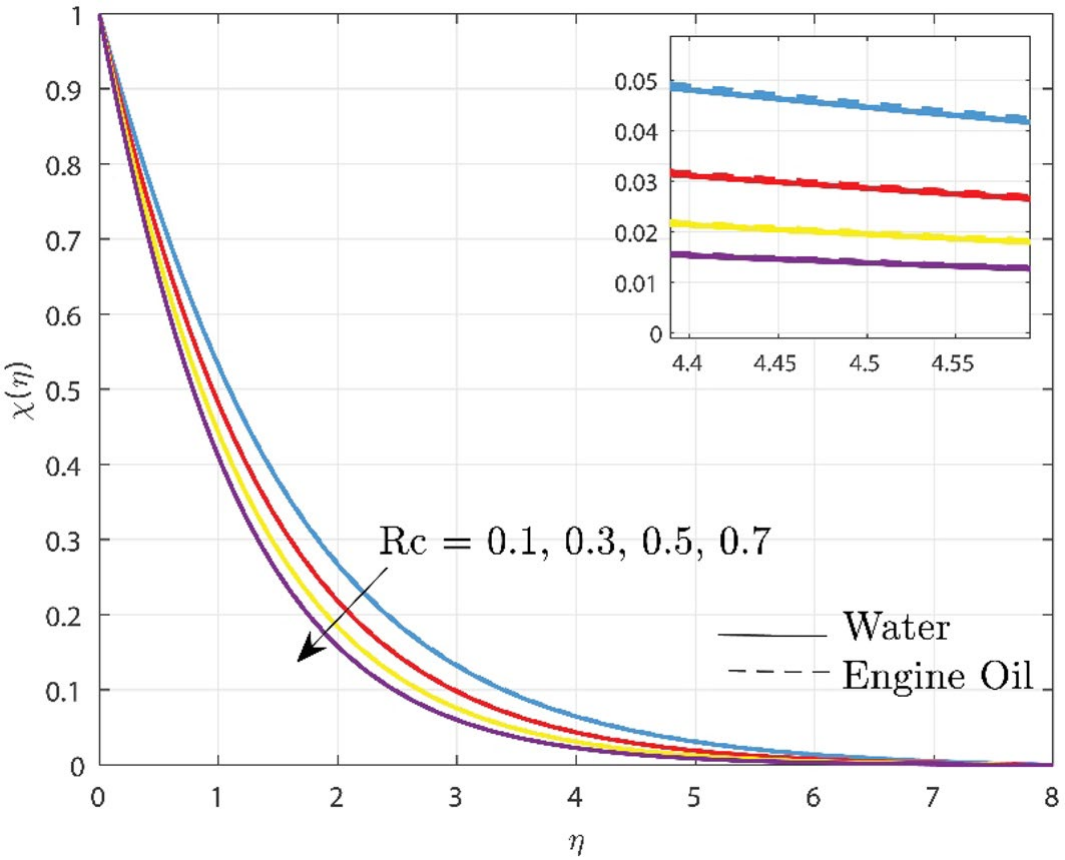
Upshot of $Rc$ over concentration profile.

Variations in $\chi \left( \eta \right)$ for different values of reaction rate parameter is observed in Fig. 5. The main reason for this is that when the $Rc$ grows, the number of solute molecules undergoing chemical reaction gets bigger, resulting in a drop in the concentration field. As a result, a damaging chemical reaction dramatically diminishes the solutal boundary layer thickness. Due to the variations in their chemical characteristics, chemical reactions in engine oil tend to proceed more slowly than in water. Hence the reduction in concentration is seen more in water than in engine oil.

**Figure 6:**
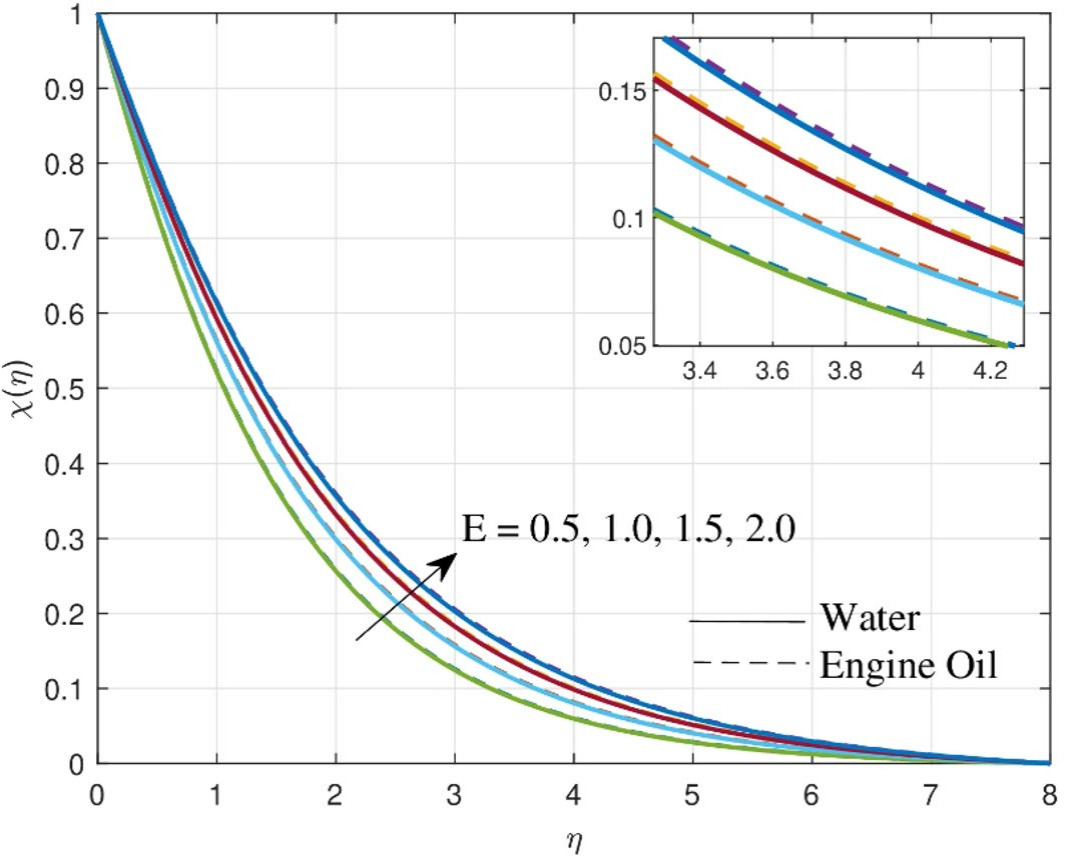
Upshot of $E$ over concentration profile.

Figure 6 reveals the physical effect of activation energy $E$ on $\chi \left( \eta \right)$. Larger values of $E$ augments the $\chi \left( \eta \right)$. The Arrhenius function deteriorates as the activation energy value increases rapidly, resulting in a stimulation of the generative chemical reaction and an improvement in the concentration field. Because of the differences in their chemical properties, elevations in concentration are witnessed more efficiently in engine oil than in water. In thermal research, activation energy is used to determine how much energy is required for molecules to shift from one state to another. This information helps to improve temperature-dependent reactions and thermal management systems.

**Figure 7:**
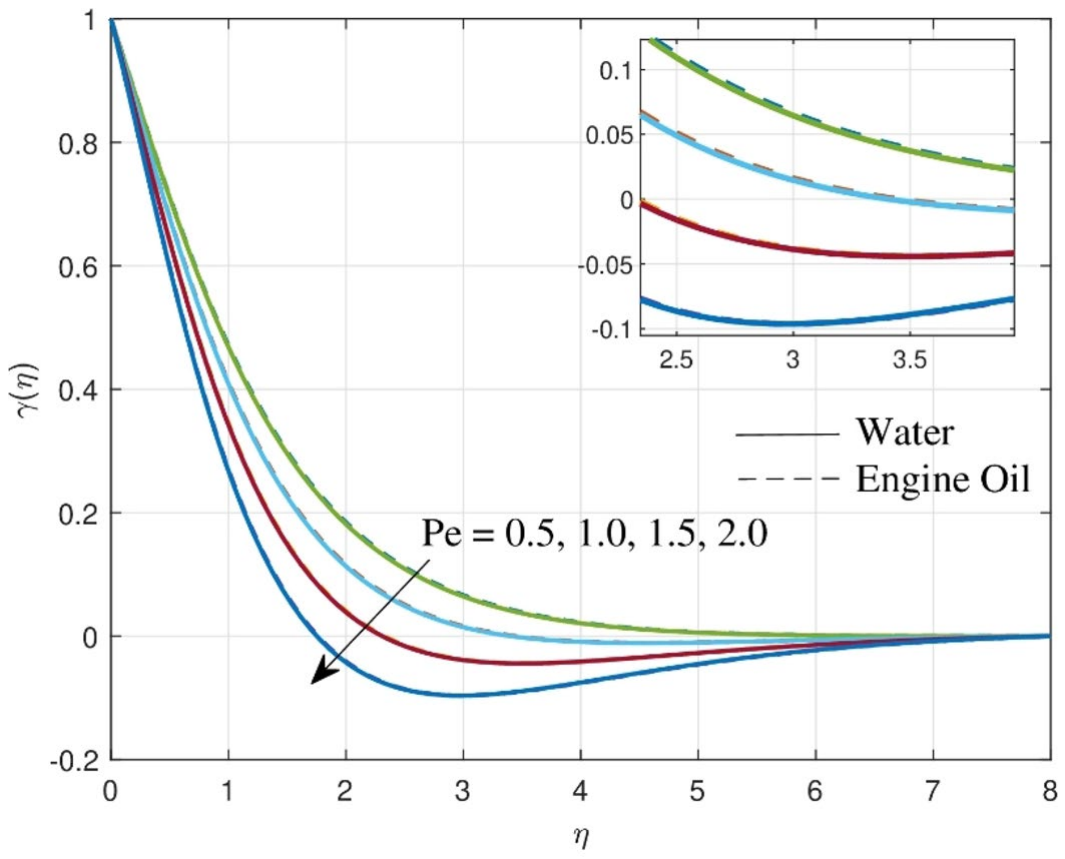
Consequence of $Pe$ over motile density profile.

The effect of Peclet number $\left( {Pe} \right)$ against motile density profile $\gamma \left( \eta \right)$ is portrayed in Fig. 7. The figure indicates that fostering $Pe$ results in microbe density condensing. Physically, a faster rate of advective movement results in a greater Peclet number, which rapidly increases the flux of microorganisms. Considering the Peclet number is inversely related to cell swimming speed, boosting the Peclet number can result in a drop in the density profile of motile microbes.

**Figure 8:**
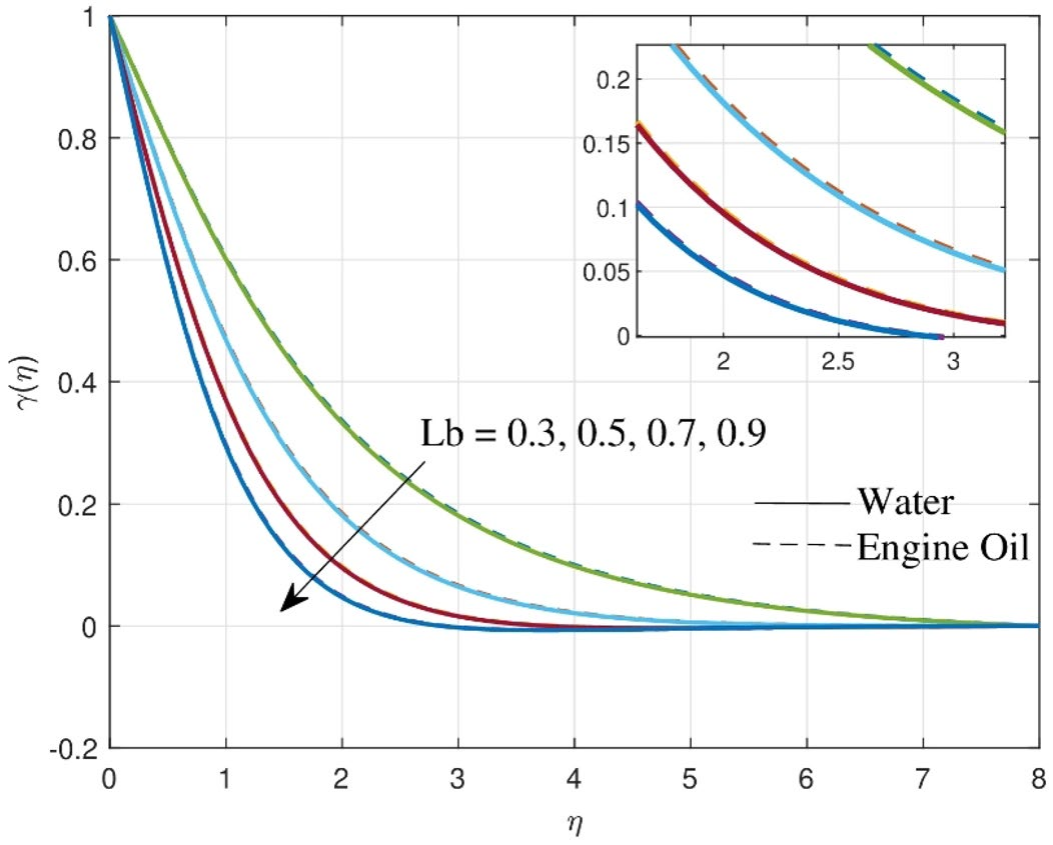
Consequence of $Le$ over motile density profile.

Figure 8 exhibits the impact of Lewis number $\left( {Lb} \right)$ on $\gamma \left( \eta \right)$. Escalating the values of $Lb$ tends to diminish the $\gamma \left( \eta \right)$. From a physical point of view in a bioconvection flow scenario, the $Lb$ regulates the behavior and diffusivity of microorganisms. A rise in the bioconvection Lewis number causes microbe diffusivity to go down resulting in a reduction in their profile. Bioconvection happens in both water and engine oil, but its factors and implications differ, therefore water has a greater decline in motile density than engine oil.

**Figure 9:**
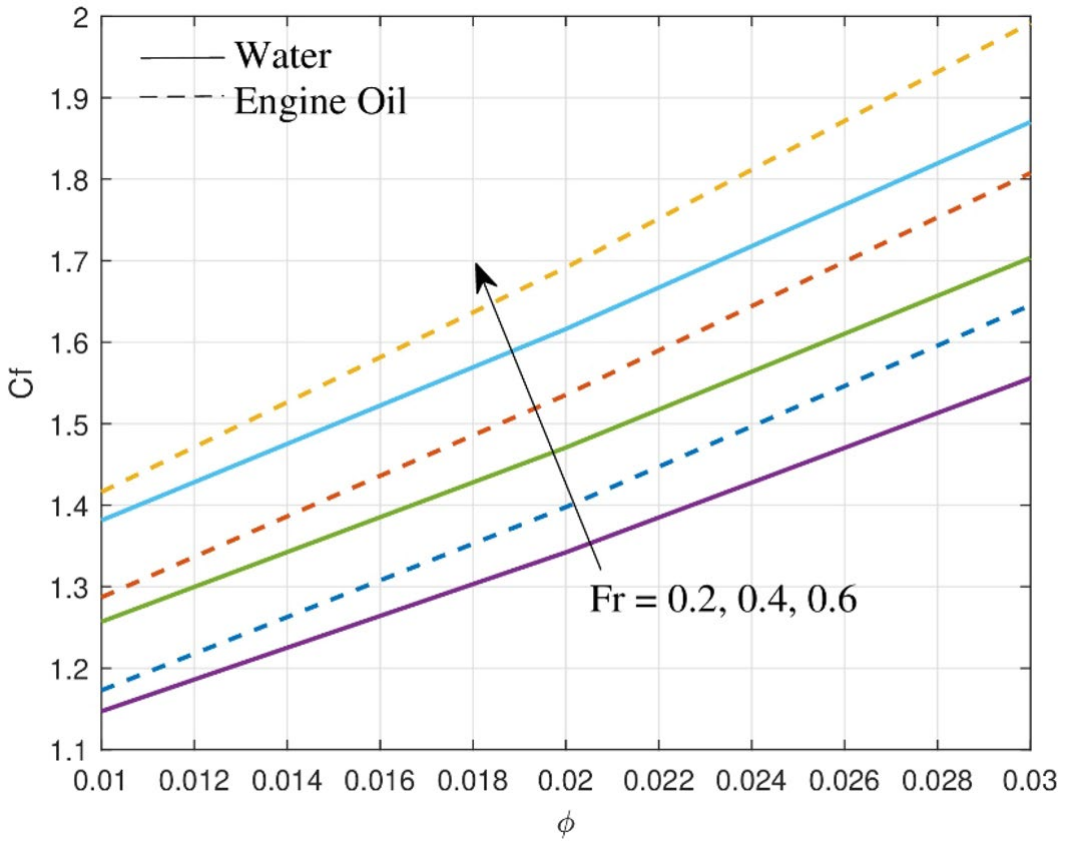
Outcome of $Cf$ over skin-friction.

The Darcy–Forchheimer model is critical for optimizing thermal management systems. For higher values of the Forchhiemer number, the skin-friction coefficient increases, influencing the efficiency of the heat transmission process. Figure 9 provides useful insights into the effect of $Fr$ on skin friction $Cf$ for two different base fluids water and engine oil. From a physical perspective, the inertia coefficient is directly proportional to the porosity of the medium and the drag coefficient, as $Cb$ grows, so does the porosity of the medium and the drag coefficient. As a result, the $Cf$ is increased, resulting in a decreased velocity. Because of their physical qualities, engine oil has a greater increase in the $Cf$ than water.

**Figure 10:**
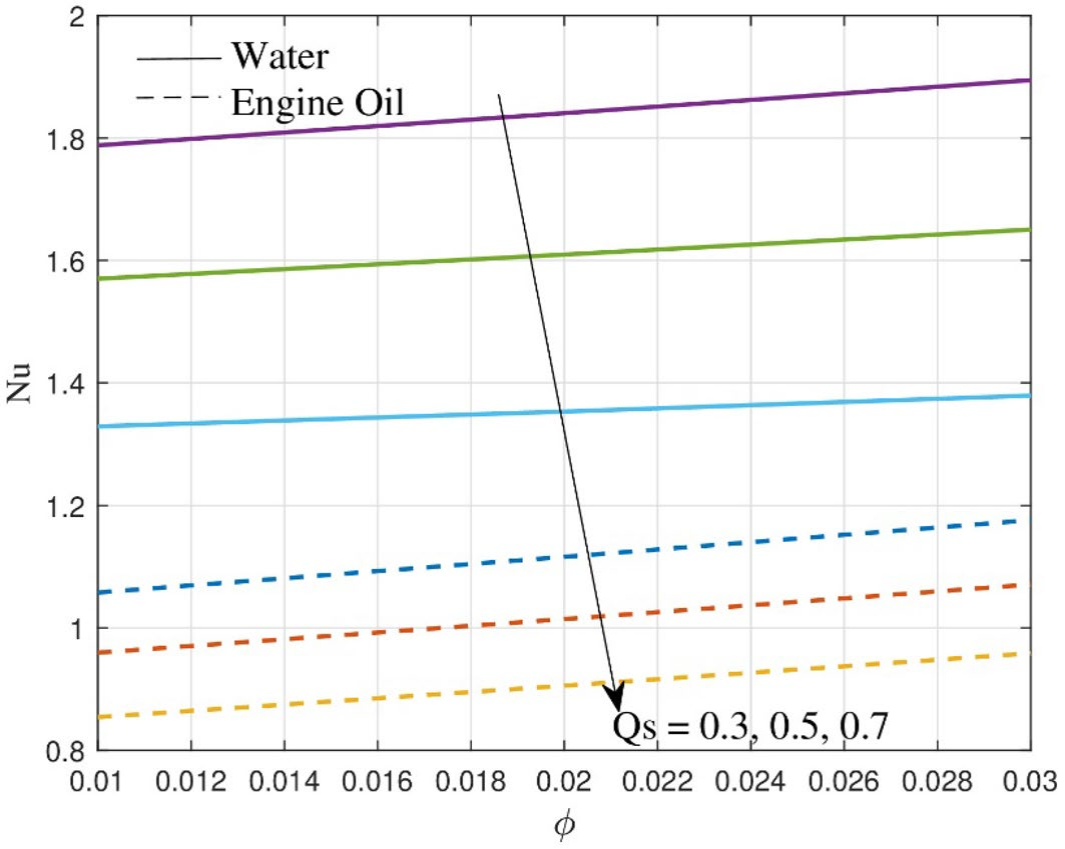
Outcome of $Nu$ over $Qs$.

In some industrial processes, particularly those that require precise control over heat transfer rates, where excessive heat transfer is undesirable or to avoid overheating, minimizing the Nusselt number can help improve energy efficiency by eliminating unnecessary heat loss. Figure 10 provides considerable details on the contrast between the exponential heat source/sink $Qs$ and $Nu$ profile in the context of a ternary nanofluid flow, where the base fluids are engine oil and water. The $Nu$ is estimated to decrease as the $Qs$ goes up. The $Nu$ drops because an increase in the aforementioned parameter induces a rise in the thermal boundary layer structure, resulting in less heat transfer rate. From the figure, it is very clear that the drop is significant in engine oil.

**Figure 11:**
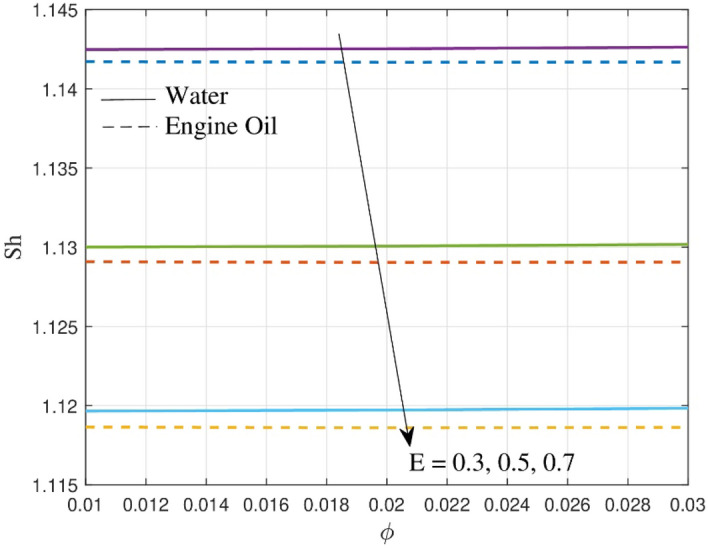
Outcome of $Sh$ over Sherwood number.

The impact of activation energy $\left( E \right)$ on $Sh$ is demonstrated in Fig. 11. It is clear from the figure that $Sh$ curves rise for augmented values of $E$. From the physical point of view, the parameter $E$ can influence the concentration distribution and mass transfer rate, which are able to alter the Sherwood number. A lower mass transfer rate, and hence a lower Sherwood number, could occur from a larger $E$ values. The impact of $E$ on concentration in engine oil decreases faster than in water due to differences in their chemical properties. The drop in the Sherwood number as activation energy grows reveals that convective mass transfer becomes more dominant than diffusion, resulting in modifications to the system's mass transfer characteristics.

**Figure 12:**
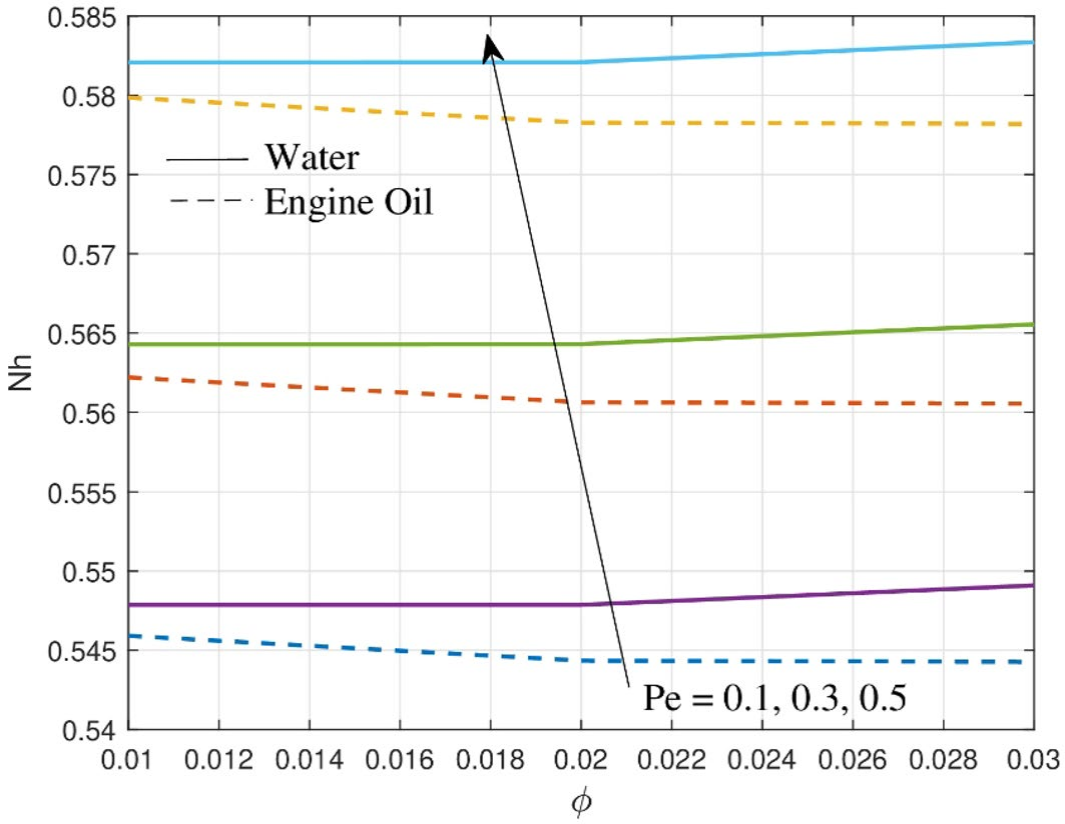
Outcome of $Nh$ over motile density profile.

How Peclet number $Pe$ affects density motile microbes $\left( {Nh} \right)$ is demonstrated in Fig. 12. The flux of wall motile microorganisms increases as $Pe$ increases. From physical point of view, the impact of bioconvection $Pe$ intensifies the swimming rate of motile microorganisms, which reduces the thickness of the microorganisms near the sheet surface thereby intensifying $Nh$ profile. Both water and engine oil undergo bioconvection, but because of the different mechanisms and adverse effects in this process, water has a larger raise in motile density than engine oil. The Peclet number has significance for determining motile density profiles and influencing mass transport rates in industrial applications, especially in bioconvection research and nanofluid flows through porous media.

## Concluding remarks

Current research incorporates a novel study of TNF across a Darcy–Forchheimer medium over a radially stretching sheet in the presence of gyrotactic microorganisms. The flow is also accomplished by exponential heat source/sink and activation energy. This study offers a comparative heat and mass transfer analysis between two base fluids engine oil and water which provides valuable data that engine oil excels in lubrication and heat dissipation for machinery whereas water is often used in cooling systems and in steam generations. The bvp4c approach is used for tackling the reduced ODEs. The major outcomes of the current problem are:

- The fluid velocity reduces with increasing $Fr$ values but opposite trend is witnessed in case of temperature profile.
- The increasing exponential heat source/sink $Qs$ boosts heat transfer inside the fluid, resulting in a temperature gain.
- Higher activation energy accelerates $\chi \left( \eta \right)$ altering the overall dynamics of chemical processes in the system whereas opposite trend is witnessed in $\chi \left( \eta \right)$ when $Rc$ values are rised.
- An increase in both Peclet number and Bioconvection Lewis number improves the density profile of motile microbes owing to factors such as better advection and transport mechanisms within the fluid, leading to a more concentrated dispersion of bacteria.
- The drag coefficient is enhanced by growing values of $Fr$.
- Reduced heat transfer was perceived when $Qs$ values were increased.
- The motile microorganism density number is improved by enlarged values of $Pe$.

We were able to successfully interpret Darcy Forchiemer's medium effect over ternary nanofluid flow with exponential heat source/sink and activation energy across a radially expanding sheet using computational methods. This research could be developed to incorporate other generalized non-Newtonian fluid models, CNTs, Hall effects, and stagnation point flow over various geometries.

## Data Availability

The datasets generated and/or analyzed during the current study are available from the corresponding author on reasonable request.

## Appendix

The numerical code for validation of $f^{\prime\prime}\left( 0 \right)$, $Fr = 1$, and in the absence of $A_{1} \,\,\& A_{2} \,$ is illustrated below as follows:

x = linspace(0,8,100);

yinit = zeros(1,9);

solinit = bvpinit(x, yinit);

ode = @(x,y) [y(2); y(3); 2*y(1)*y(3)-Fr*y(3)^2];

bc = @(ya,yb) [ya(1), ya(2)-1, yb(2), ya(4)-1, yb(4), ya(6)-1, yb(6), ya(8)-1, yb(8)];

sol = bvp4c(ode, bc, solinit);

disp(sol.y(2,:)).

## List of symbols

$$K_{b}$$: Boltzmann constant
$$E$$: Activation energy $$({\text{kg}}\;{\text{m}}^{2} \;{\text{s}}^{ - 2} )$$
$$C$$: Concentration $$({\text{mol}}\;{\text{m}}^{ - 3} )$$
$C_{w}$: Concentration at wall $({\text{mol}}\;{\text{m}}^{ - 3} )$
$$C_{\infty }$$: Ambient concentration $$({\text{mol}}\;{\text{m}}^{ - 3} )$$
$$b$$: Chemotaxis constant $$({\text{m}})$$
$$a$$: Constant
$$A_{1} ,A_{2} ,A_{3} ,A_{4}$$: Constants
$k_{r}$: Chemical reaction parameter $({\text{s}}^{ - 1} )$
$Qs$: Exponential heat source/sink parameter
$Q^{*}$: Dimensional heat generation/absorption coefficient $({\text{W}}\;{\text{m}}^{ - 3} \;{\text{K}}^{ - 1} )$
$$Fr$$: Forchheimer number
${\text{Re}}$: Local Reynolds number
$Lb$: Bio-convection Lewis number
$W_{c}$: Maximum cell swimming speed
$D_{N}$: Microorganisms diffusion coefficient
$N_{w}$: Motile microorganisms at the surface
$N_{\infty }$: Motile organisms at the far field
$$Nu$$: Nusselt number
$Nh$: Microorganism’s density number
$${\text{Pe}}$$: Peclet number
$$\Pr$$: Prandtl number
$$\left( {r,\,z} \right)$$: Polar-coordinates (m)
$Rc$: Reaction rate parameter
$Cf$: Skin-friction coefficient
$$Sh$$: Sherwood number
$$Sc$$: Schimdt number
$Cp$: Specific heat capacity ${\text{(J}}\;{\text{kg}}^{ - 1} \;{\text{K}}^{ - 1} )$
$$T$$: Temperature (K)
$$T_{w}$$: Wall temperature (K)
$T_{\infty }$: Temperature at far-field (K)
$$k$$: Thermal conductivity $$({\text{kg}}\;{\text{m}}\;{\text{s}}^{ - 3} \;{\text{K}}^{ - 1} )$$
$u_{w}$: Uniform radial velocity $({\text{m}}\;{\text{s}}^{ - 1} )$
$$\left( {u,w} \right)$$: Velocity components $$({\text{m}}\;{\text{s}}^{ - 1} )$$
$$f^{\prime}\left( \eta \right)$$: Velocity profile

## Greek symbols

$$\rho$$: Density $$({\text{kg}}\;{\text{m}}^{ - 3} )$$
$$\mu$$: Dynamic viscosity $$({\text{kg}}\;{\text{m}}^{ - 1} \;{\text{s}}^{ - 1} )$$
$$\nu$$: Kinematic viscosity $$({\text{m}}^{2} \;{\text{s}}^{ - 1} )$$
$\alpha$: Base fluid’s thermal diffusivity $({\text{m}}^{2} \;{\text{s}}^{ - 1} )$
$$\theta \left( \eta \right)$$: Temperature profile
$$\chi \left( \eta \right)$$: Mass concentration
$\gamma \left( \eta \right)$: Motile density profile
$\phi_{1} ,\phi_{2} ,\phi_{3}$: Solid volume fractions of ternary nanoparticles
$$\eta$$: Similarity variable
$$\delta$$: Temperature difference

## Abbreviations

ODEs: Ordinary differential equations
PDEs: Partial differential equations
MHD: Magnetohydrodynamics
CNTs: Carbon nano tubes
$$\begin{gathered} {\text{AA7072}},\, \hfill \\ {\text{AA7075}} \hfill \\ \end{gathered}$$: Aluminium alloys

## Subscripts

$$f$$: Base fluid
$$nf$$: Nanofluid
$$hnf$$: Hybrid nanofluid
$$tnf$$: Ternary nanofluid
